# Rational design of a cyclohexanone dehydrogenase for enhanced α,β-desaturation and substrate specificity†

**DOI:** 10.1039/d3sc04009g

**Published:** 2024-02-21

**Authors:** Warispreet Singh, Nicola L. Brown, Hannah V. McCue, Sophie R. Marriott, Richard C. Wilson, Justin Perry, Johan P. Turkenburg, Kshatresh D. Dubey, Stephen H. Prior, Andrew J. Carnell, Edward J. Taylor, Gary W. Black

**Affiliations:** a Hub for Biotechnology in Build Environment, Department of Applied Sciences, Faculty of Health and Life Sciences, Northumbria University Newcastle upon Tyne NE1 8ST UK gary.black@northumbria.ac.uk; b Institute of Systems, Molecular and Integrative Biology, University of Liverpool Crown Street Liverpool L69 7ZB UK; c Department of Life Sciences, University of Lincoln Lincoln LN6 7TS UK etaylor@lincoln.ac.uk; d Structural Biology Laboratory, Department of Chemistry, University of York YO10 5DD UK; e Department of Chemistry, Center for Informatics, School of Natural Sciences, Shiv Nadar University Uttar Pradesh-201314 India; f School of Chemistry, University of Lincoln Lincoln LN6 7DL UK; g Department of Chemistry, University of Liverpool Crown Street Liverpool L69 7ZD UK

## Abstract

The selective α,β-desaturation of cyclic carbonyl compounds, which are found in the core of many steroid and bioactive molecules, using green chemistry is highly desirable. To achieve this task, we have for the first time described and solved the *de novo* structure of a member of the cyclohexanone dehydrogenase class of enzymes. The breadth of substrate specificity was investigated by assaying the cyclohexanone dehydrogenase, from *Alicycliphilus denitrificans*, against several cyclic ketones, lactones and lactams. To investigate substrate binding, a catalytic variant, Y195F, was generated and used to obtain a crystallographic complex with the natural substrate, cyclohexanone. This revealed substrate–active site interactions, as well as the proximity of the cofactor, flavin adenine dinucleotide, and enabled us to propose a mechanistic function to key amino acids. We then used molecular dynamic simulations to guide design to add functionality to the cyclohexanone dehydrogenase enzyme. The resulting W113A variant had overall improved enzyme activity and substrate scope, *i.e.*, accepting the bulkier carbonyl compound, dihydrocoumarin. Structural analysis of the W113A variant revealed a broader, more open active site, which helped explain the modified substrate specificity. This work paves the way for future bespoke regioselective α,β-desaturation in the synthesis of important bioactive molecules *via* rational enzyme engineering.

## Abstract 2

The W113A cyclohexanone dehydrogenase variant had improved substrate scope, *i.e.*, accepting a bulkier compound, compared to wildtype enzyme. Structural analysis of the variant revealed a broader, more open active site, explaining the new substrate scope.

## Graphical abstract

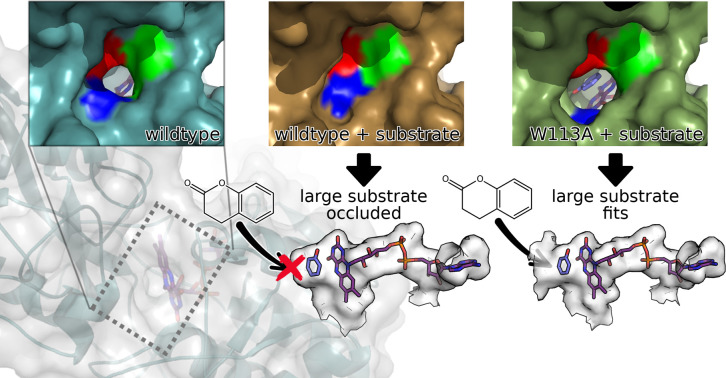

## Introduction

**Scheme 1:**
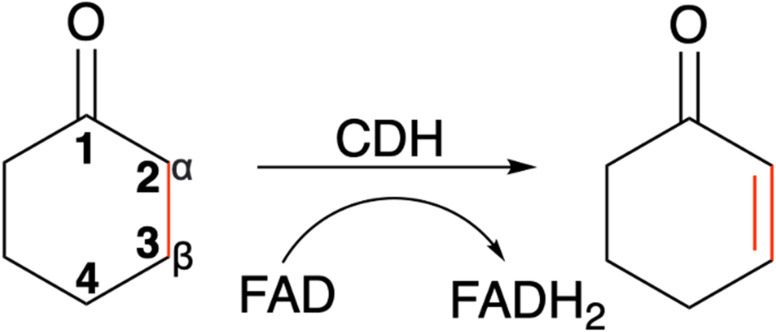
The α,β-desaturation of cyclohexanone to 2-cyclohexen-1-one catalysed by cyclohexanone dehydrogenase (CDH) in the presence of flavin adenine dinucleotide (FAD), which is reduced to FADH_2_.

The enantio- and regioselective dehydrogenation of cyclic ketones, which are found in the core of many steroid and bioactive molecules, is a challenging task synthetically.^1,2^ The conventional approaches for selective α,β-desaturation of cyclic ketones requires the use of highly toxic reagents and strong oxidants. Even recently developed palladium and platinum transition metal catalysts, which work under much milder conditions in comparison to their traditional counterparts, are still harmful to the environment.^3^ Moreover, the use of the above approaches for ketone desaturation lacks enantio- and regioselective control. Additionally, the final product is often contaminated with side products due to over oxidation and therefore requires considerable purification efforts. This manuscript focuses on a cyclohexanone dehydrogenase (CDH), EC number 1.3.99.14, an enzyme which can perform α,β-desaturation of carbonyl compounds with exquisite selectivity. The natural substrate for CDHs is cyclohexanone, a simple cyclic ketone. Interestingly, although CDH activity was observed in a cell extract of a denitrifying pseudomonad in 1989,^4^ the enzyme has never been identified and characterised until now. CDHs belongs to the succinate dehydrogenase/fumarate reductase flavoprotein superfamily and use flavin adenine dinucleotide (FAD) as a cofactor to perform the dehydrogenation of cyclic ketones (Scheme 1). Another example of an enzyme, which belongs to the succinate dehydrogenase/fumarate reductase flavoprotein superfamily, is 3-ketosteroid Δ^1^-dehydrogenase (KSTD). This enzyme catalyses the regioselective α,β-desaturation of steroids and has high importance in the pharmaceutical industry for the synthesis of steroid drugs. Another recent study by the same group also identified another KSTD enzyme from *Sterolibacterium denitrificans*.^5^ The Δ^1^-dehydrogenation reaction catalysed was studied for eight different steroid molecules and origin of substrate selectivity and catalytic mechanism were in accordance with their previous work.^6^ These studies indicate the importance of KSTDs in the synthesis of various steroid drugs.^5–7^ Therefore, the development of a new class of enzyme which will perform enantio- and regioselective dehydrogenation of cyclic ketones, which are found in the core of many steroid molecules, will be an attractive and greener approach for the pharmaceutical industry.

To make CDH a more versatile catalyst for the synthesis of various bioactive molecules, we have presented in this manuscript the *de novo* X-ray structure of the CDH from *Alicycliphilus denitrificans* in complex with cyclohexanone, and rational design of the enzyme, using molecular dynamics (MD) simulations, to improve its substrate scope and enhance the dehydrogenation of cyclic ketones. The overall aim of this project was to identify an enzyme with CDH activity, investigate the active site layout and define key active site interactions with the cyclohexanone ligand. Using the defined structural parameters and modelling software we were then able to predict mutations that may result in modified substrate specificity. The effects of the predicted mutations were tested and confirmed using biochemical analysis and rationally explored using structural biology.

## Methods

### Cloning and mutagenesis of the cdh gene

Identification of a gene encoding CDH was facilitated due to its proximity to the three genes encoding the characterised 3-hydroxycyclohexanone dehydrogenase (3-HCDH) from *Alicycliphilus denitrificans* K601,^8^ an enzyme, composed of three different subunits, that takes the hydrated product of CDH and converts it to 1,3-cyclohexanedione. The gene (ENA|AEB86630) upstream of the genes encoding the three different 3-HCDH subunits was amplified from a glycerol stock of *Alicycliphilus denitrificans* (strain DSM 14773/CIP 107495/K601) using Q5 Hot Start High-Fidelity 2X Master Mix (New England Biolabs) in accordance with the manufacturer's instructions with the following primers 5′TGCCATAGGAATTCATGATGACGACTAAACCTAATGCC3′ and 5′TGCCATAGAAGCTTCTAGGTACGCTTGGGTTCG3′ (Integrated DNA Technologies) and ligated into pET28a (Novagen) on a *Eco*RI–*Hin*dIII fragment, so that CDH was encoded with a N-terminal hexahistidine tag for ease of purification. The sequence of the insert of the generated construct, p-*cdh*, was confirmed by Sanger sequencing (MRC PPU DNA Sequencing and Services).

### Generation of Y195F and W113A mutant constructs

Mutants were generated using the QuikChange site-directed mutagenesis kit (Agilent) in accordance with the manufacturer's instructions using the p-*cdh* construct as a template with the following primer pairs (Y195F_FP CCCTTCGCCACAGACTTTTTCTCTGAACTGCCC; Y195F_RP GGGAAGCGGTGTCTGAAAAAGAGACTTGACGGG; W113A_FP AACGTTCAAATCAGCATTCGCGTACTGGGTGCCGAACAAT; W113A_RP TTGCAAGTTTAGTCGTAAGCGCATGACCCACGGCTTGTTA). The resulting constructs were termed p-*cdhY195F* or p-*cdhW113A*, respectively. The sequence of the inserts of p-*cdhY195F* or p-*cdhW113A* was confirmed by Sanger sequencing.

### Protein expression and purification

For the expression of the WT CDH and variant CDH proteins, p-*cdh*, p-*cdhY195F* or p-*cdhW113A* constructs were transformed into *Escherichia coli* BL21(DE3) (Novagen). Cultures were grown in 0.5 L of LB media in a 2 L baffled flask at 37 °C and shaken at 180 rpm to an OD of 1.0 *A*_600nm_. The temperature was reduced to 30 °C prior to induction with IPTG at a final concentration of 1 mM and incubated overnight (16 h). For the expression of the selenomethionine (SelenoMet) wild type (WT) CDH, the p-*cdh* construct was transformed into *E. coli* B834(DE3) (Novagen). Cultures were grown in SelenoMet Medium (Molecular Dimensions) in accordance with the manufacturer's instructions. The temperature was reduced to 30 °C prior to induction with IPTG at a final concentration of 1 mM and incubated overnight (16 h). In all cases cells were harvested by centrifugation (15 min, 4000 × *g*, 4 °C), resuspended in 5 mL of 50 mM Na_2_HPO_4_–HCl, 0.5 M NaCl, 10 mM imidazole, pH 7.4 followed by cell disruption by sonication (6 × 10 s at 14 microns). All CDH proteins were purified from cell free extracts *via* affinity chromatography using Ni-affinity chromatography followed by gel filtration. N-terminally hexahistidine tagged protein products were purified *via* immobilised metal ion (Ni^2+^) chromatography (Cytiva) using a linear gradient of 10 to 500 mM imidazole.

### Crystallization and structure solution

Pure proteins, as judged by SDS-PAGE, were concentrated to between 5–10 mg mL^−1^ and buffer exchanged into 20 mM Tris–HCl, pH 7.2 using a Vivaspin 10 kDa cut-off concentrator. The proteins were screened in 300 nL drops using a mosquito crystallization robot (SPT Labtech) together with the Hampton Crystal Screen, Crystal Screen 2 and Hampton PEG/Ion Screens I and II (Hampton Research). Conditions were optimised manually using the hanging drop vapour diffusion method. Drops containing 1 μL or 2 μL of protein were mixed with 1 μL of the mother liquor. Initial crystals were found to grow in Hampton PEG/Ion Screen I condition 2 and 48. This condition was optimised further to improve crystal quality. Best crystals were obtained in a 0.24 M ammonium phosphate dibasic, 20% PEG 3550 solution or a 0.11 M ammonium citrate dibasic 12% PEG 3550. A cryo-protectant solution was produced by supplementing the mother liquor with an additional 25% glycerol. The crystals formed and were harvested in rayon fibre loops, bathed in cryo-protectant solution prior to flash freezing in liquid nitrogen. To obtain cyclohexanone complexes, crystals were grown from the Y195F variant in 0.11 M ammonium citrate dibasic 12% PEG 3550 supplemented with cyclohexanone at a final concentration of 10 mM.

Data were collected on the Diamond Light Source from a single crystal at 100 K with an oscillation/rotation range of 0.10°. SelenoMet WT CDH data were collected at a wavelength of 0.9786 Å over an oscillation/rotation range of 720° on I24 using a Pilatus3 6M detector. The data were processed with XDS.^9^ The crystals displayed *P*2_1_2_1_2 space group symmetry with the approximate cell dimensions of *a* = 158.91 Å, *b* = 66.71 Å, *c* = 121.91 Å, with two predicted molecules in the asymmetric unit at a solvent content of 47%. The structure solution was determined automatically using the *crank2* – automated structure solution pipeline for SAD.^10^ A test set of 5% of reflections was defined for the calculation of *R*_free_ for cross validation before model building. This set was used to monitor the module at various stages of refinement for the weighting of geometrical and temperature factor restraints. REFMAC^11^ in conjunction with BUCCANEER^12^ were used to build the sequence into the electron density automatically. Solvent molecules were added using COOT^13^ and checked manually.

Higher resolution native data were collected at a wavelength of 0.9726 Å over an oscillation/rotation range of 220° on I04 using a Pilatus3 6M detector. The data were processed with Xia2.^14^ The crystals displayed *P*2_1_2_1_2 space group symmetry with different approximate cell dimensions of *a* = 67.0 Å, *b* = 120.3 Å, *c* = 176.1 Å, with two predicted molecules in the asymmetric unit at solvent content of 51%. The high-resolution structure was solved using the partially refined model built using the selenium data and solved using MOLREP.^15^ The native model was built using BUCCANEER^12^ in conjunction with REFMAC^11^ and COOT^13^ and checked manually. The structure was validated^16^ prior to deposition. All other computing was undertaken using the CCP4 suite^17^ unless otherwise mentioned.

### Biochemical analysis

**Fig. 1:**
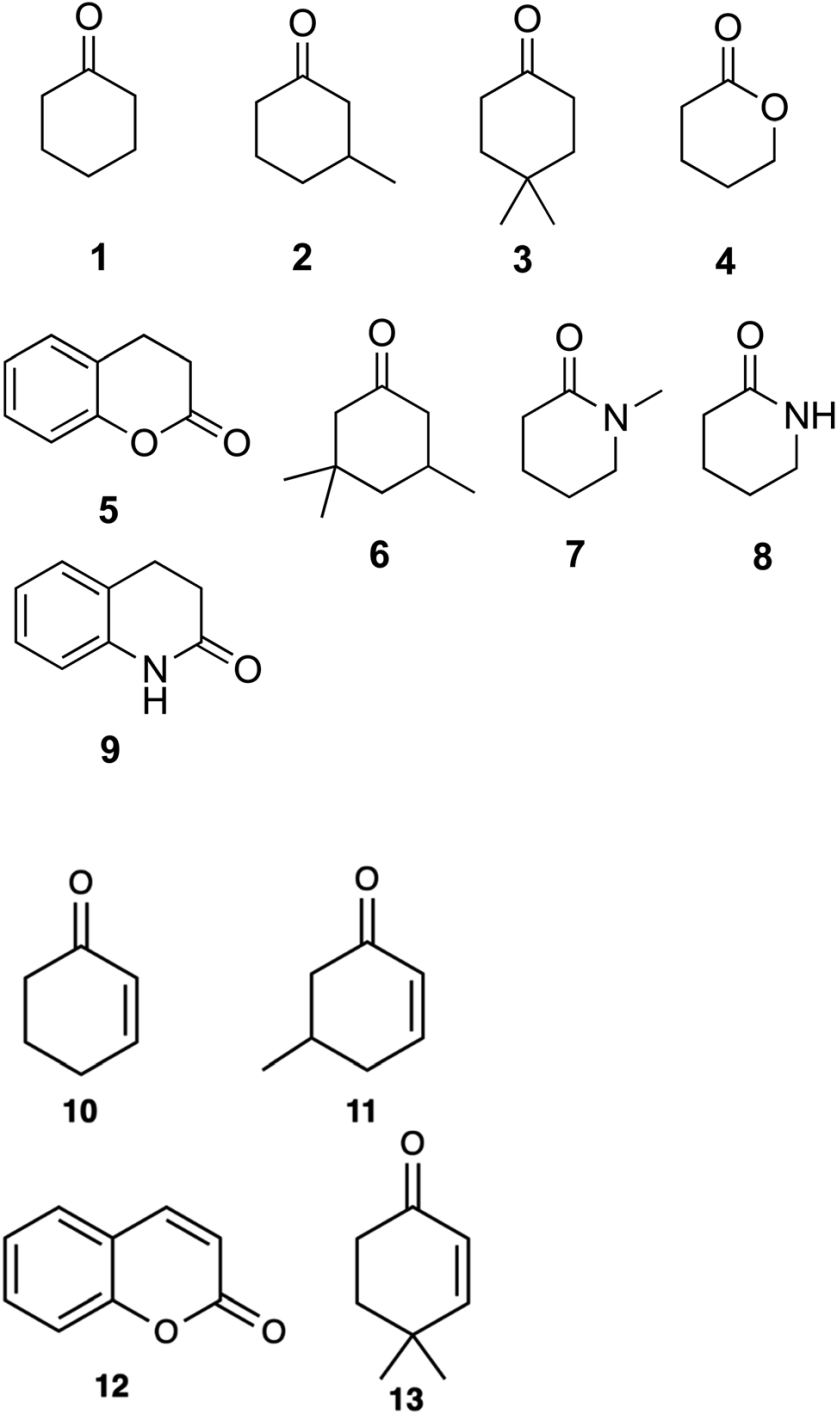
Cyclic substrates tested for α,β-desaturation by WT-CDH and W113A mutant: cyclohexanone (1); 3-methylcyclohexanone (2); 4,4-dimethylcyclohexanone (3); δ-valerolactone (4); dihydrocoumarin (5); 3,3,5-trimethylcyclohexanone (6); 1-methyl-2-piperidone (7); δ-valerolactam (8); 3,4-dihydro-2(1*H*)-quinolinone (9). Products obtained from the α,β-desaturation by WT-CDH and W113A mutant: 2-cyclohexen-1-one (10); 5-methyl-2-cyclohexen-1-one (11); coumarin (12); isophorone (13).

Substrate scoping of WT CDH and the two variants was performed in a 96-well microtitre plate at 578 nm over 5 min with 0.25 μM enzyme, 2 mM substrate and 75 μM 2,6-dichlorophenol-indophenol (DCPIP) in 20 mM Tris–HCl pH 7.8. Several cyclic ketones, lactones and lactams were tested (Fig. 1). Kinetics of WT CDH was performed with 76 nM enzyme (for cyclohexanone, 3-methylcyclohexanone and 4,4-dimethylcyclohexanone), 60 μM DCPIP in 20 mM Tris–HCl pH 7.8 and varying concentrations of each substrate (20 mM, 15 mM, 10 mM, 5 mM, 2.5 mM, 1.25 mM, 0.625 mM, 0.3125 mM).

Product identification was performed using HPLC. Samples were run on a Hypersil Gold aQ column. The mobile phase was 9%/91% isopropanol/100 mM ammonium acetate pH 4.5 (isocratic). The column was maintained at 40 °C and samples were stored in the autosampler at 4 °C. Chromatograms were recorded at 240 nm and 275 nm. Reactions contained 1 μM enzyme, 1 mM substrate and 100 μM DCPIP in 20 mM Tris–HCl buffer pH 7.8. The reactions were left at room temperature for 1 hour and then stopped using the addition of 100 μL acetic acid.

NMR spectra were acquired on a Bruker Avance III HD spectrometer operating at a proton Larmor frequency of 500 MHz, using a room temperature broadband probe. Spectra were acquired at 298.15 K. Spectra were acquired using Noesy presaturation of solvent peaks and with 128k complex points and 128 transients.

### Computational methods

Parameterization for the various substrates and covalently linked FAD molecule to the Cys305 residue of the CDH enzyme were developed using the general Amber force field (GAFF)^18^ using Antechamber module available in AmberTools20.^19^ The partial charges for the ligands were calculated by running quantum mechanics calculation at HF/6-31G* level of theory using the restrained electrostatic potential (RESP) method in Gaussian 16 ^20^ package. Flexible ligand docking of cyclohexanone, 3-methylcyclohexanone, dimethylcyclohexanone and dihydrocoumarin were performed using the energy minimised X-ray structures of WT CDH and the W113A variant using AutoDock 4.2 suite with the Lamarckian genetic algorithm (LGA).^21^ The grid box was centred around the active site pocket consisting of Y195, Y376 and FAD molecule and total of 300 LGA runs were carried out for each protein ligand complex for both the WT CDH and the W113A variant.

The production MD simulations were performed using the graphics processing unit (GPU) version of particle mesh Ewald molecular dynamics (PMEMD) integrated with Amber 20.^19,22,23^ The protonation states of the titratable residues were predicted using the H++ server.^24^ The FF19SB force field^25^ was employed in all the simulations and Leap module was used to add the five chloride ions to neutralise the protein system. Each of the enzyme complex systems were immersed into a truncated octahedral box of TIP3P^26^ water molecules with the boundary of protein system being 10 Å away from the box edges. The periodic boundary conditions were employed in all the simulations. Long-range electrostatic interactions were calculated using the particle mesh Ewald (PME)^27^ with a cut-off of 8 Å for the direct space Coulomb and vdW forces.

The solute molecules were restrained using a potential of 5 kcal mol^−1^ Å^−2^ and the solvent and ions were subjected to energy minimization (5000 steps) using steepest descent and conjugate gradient methods. The entire system was then subjected to controlled heating from 0 to 298.15 K for 50 ps at constant volume using Langevin thermostat with a collision frequency of 1/ps using a canonical ensemble. During the heating process, the non-hydrogen atoms of the solute molecules were restrained using a harmonic potential of 5 kcal mol^−1^ Å^−2^. This was followed by another round of energy minimization for 2000 steps using steepest descent and conjugate gradient methods. The entire system was then subjected to two rounds of equilibration at 298.15 K for 50 ps using a weak restrain of 0.1 kcal mol^−1^ Å^−2^ on all the solute atoms in an NPT ensemble. A Berendsen barostat was used to maintain the pressure at 1 bar and the SHAKE^28^ algorithm was used to constrain bonds involving hydrogen. A time step of 2 fs was used for all MD runs. A production MD for each system was run for continuous 500 ns was performed in an NPT ensemble with a target pressure of 1 bar and a pressure coupling constant of 2 ps. The Root Mean Square Deviation (RMSD) and cluster analysis were performed using CPPTRAJ.^29^

## Results and discussion

### Biochemical characterisation of WT CDH and the Y195F and W113A variants

**Fig. 2:**
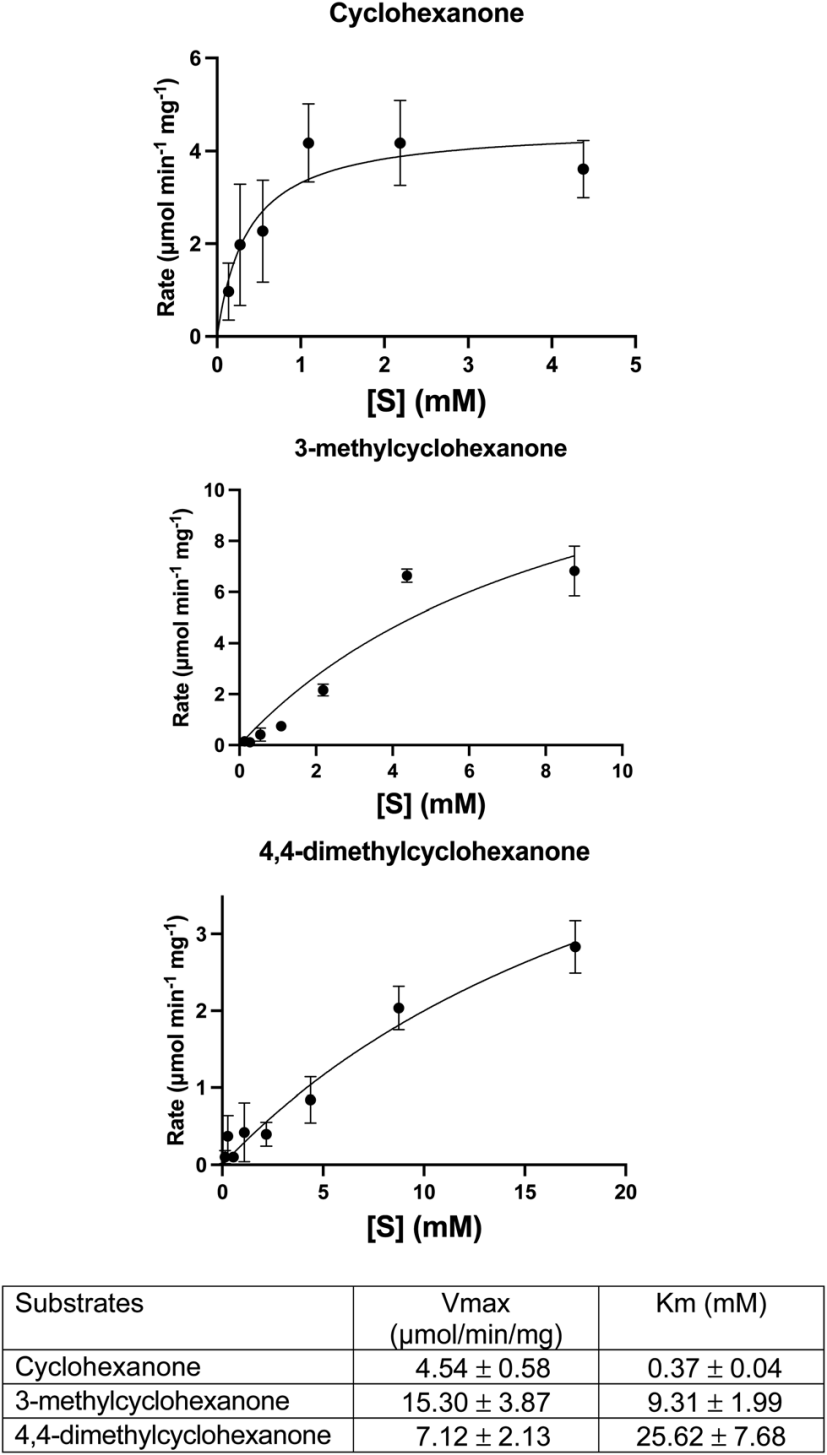
Michaelis–Menten plots and parameters for WT CDH with the following substrates: cyclohexanone, 3-methylcyclohexanone and 4,4-dimethylcyclohexanone. Error bars represent standard deviations of the values obtained from triplicate experiments. Derived parameters are the means ± standard deviations.

A DCPIP colour change assay was used initially to assess any differences in activity of WT CDH and W113A and Y195F variants on cyclohexanone and the eight other test substrates (Fig. 1). Activity was observed for (i) WT CDH and the W113A variant with cyclohexanone, 3-methylcyclohexanone and 4,4-dimethylcyclohexanone, and (ii) the W113A variant, but not WT CDH, with dihydrocoumarin (Fig. S1A†). No activity was observed for (i) WT CDH or the W113A variant with δ-valerolactone, 3,3,5-trimethylcyclohexanone, *N*-methyl-2-piperidone or 3,4-dihydro-2(1*H*)-quinolone, and (ii) the Y195F variant with any substrate (Fig. S1A†). Time course analyses using the DCPIP assay gave greater detail about the differences in substrate specificity. WT CDH showed highest activity against cyclohexanone, closely followed by 3-methyl-cyclohexanone, weak activity against 4,4-dimethyl cyclohexanone and no activity against δ-valerolactone and dihydrocoumarin (Fig. S1B†). The W113A variant showed highest activity on 3-methylcyclohexanone, followed by cyclohexanone, 4,4-dimethylcyclohexanone and dihydrocoumarin and no activity on δ-valerolactone (Fig. S1C†). The time course confirmed the inactivity of the Y195F variant with all substrates (Fig. S1D†). It should be noted that Michaelis–Menten kinetics were not available for all enzyme with all substrates and therefore differences in substrate specificity must be treated with caution. Michaelis–Menten plots and parameters for WT CDH with cyclohexanone, 3-methylcyclohexanone and 4,4-dimethylcyclohexanone can be seen in Fig. 2. It should be noted that, due to the low aqueous solubility of 3-methylcyclohexanone and 4,4-dimethylcyclohexanone, caution must be exercised with the interpretation of the resulting kinetic data associated with these two substrates.

HPLC analysis confirmed the identity and production of the products and the concomitant formation of reduced DCPIP (Fig. S2–S4†). For 4,4-dimethylcyclohexanone, there was an additional unidentified peak in the HPLC at rt = 3.0 min which we suspect to be the doubly unsaturated ketone (Fig. S4†). Indeed, this product would be analogous to that formed by the homologous KSTD from *Rhodococcus erythropolis*.^30^

NMR analysis revealed that it was the 5-methyl-2-cyclohexen-1-one isomer that was produced from the 3-methylcyclohexanone substrate and not the other possible isomer, 3-methyl-2-cyclohexen-1-one (Fig. S5†).

### Overall structure of WT CDH

Data collection and refinement statistics are given in Table 1.

**Table 1:** Data collection and refinement statistics

	CDH SE	CDH	CDH–Y195F·A2Q	CDH–W113A·A2Q
Synchrotron source, beamline	Diamond, i24	Diamond, I04-1	Diamond, I03	Diamond, I04-1
Wavelength (Å)	0.9786	1.6000	0.9763	0.9763
Temperature (K)	100.0	100.0	100.0	100.0
*d*_max_ − *d*_min_ (Å)	96.17–1.96 (1.99–1.96)	90.70–1.86 (1.89–1.86)	60.93–1.33 (1.37–1.33)	85.65–1.85 (1.88–1.85)
Space group	*P*2_1_2_1_2_1_	*P*4_3_2_1_2	*P*2_1_2_1_2_1_	*P*4_3_2_1_2

**Unit cell parameters** a				
*a*, *b*, *c* (Å)	66.10, 121.14, 158.16	90.70, 90.70, 278.24	66.03, 121.70, 158.15	90.05, 90.05, 277.36
*α*, *β*, *γ* (Å)	90.00, 90.00, 90.00	90.00, 90.00, 90.00	90.00, 90.00, 90.00	90.00, 90.00, 90.00
Unique reflections	91 832 (4542)	97 297 (4025)	290 848 (14 363)	98 195 (4779)
Completeness (%)	100 (100)	98.8 (83.1)	99.8 (99.7)	99.9 (99.2)
Multiplicity	26.5 (27.0)	19.7 (4.1)	13.3 (12.4)	16.2 (16.7)
Anomalous multiplicity	13.85 (13.89)	NA	NA	NA
CC_1/2_	1.0 (0.7)	1.0 (0.4)	1.0 (0.6)	1.0 (1.0)
*R*_meas_	0.415 (2.511)	0.131 (1.982)	0.150 (2.212)	0.273 (3.078)
Mean *I*/*σ*(*I*)	6.2 (1.0)	1.55 (at 1.86 Å)	1.74 (at 1.33 Å)	1.27 (at 1.85 Å)
*F*_o_, *F*_c_ correlation	0.94	0.966	0.977	0.967
*R*_work_	0.239 (0.247)	0.183 (0.342)	0.179 (0.342)	0.173 (0.293)
*R*_free_	0.283 (0.284)	0.216 (0.395)	0.198 (0.352)	0.215 (0.330)
No. protein molecules per AU	2	2	2	2
No. amino acid residues ordered/total		1075/1198	1090/1198	1078/1198

**No. non-hydrogen atoms per AU**				
Total		9446	10 600	9076
Protein		8403	8875	8222
Ligand		106	162	120

**Average *B* (Å)** b				
Total		25.1	17.4	25.8
Protein		24.3	15.6	25.3
Ligand		20.5	13.5	23.0

**RMSD from ideal geometry** c				
Bonds (Å)		0.007	0.012	0.007
Angles (°)		1.470	1.818	1.376

**Ramachandran analysis**				
In preferred regions (%)		98.2	98.5	98.5
In allowed regions (%)		1.8	1.5	1.5
In disallowed regions (%)		0.0	0.0	0.0
PDB accession code		8AM3	8AM6	8AM8

a Numbers in parenthesis correspond to the high-resolution outer shell.

b Estimated standard uncertainty, based upon *R*_free_, calculated using REFMAC.

c Calculated using validation options in COOT.

#### *De novo* crystal structure of WT CDH

**Fig. 3:**
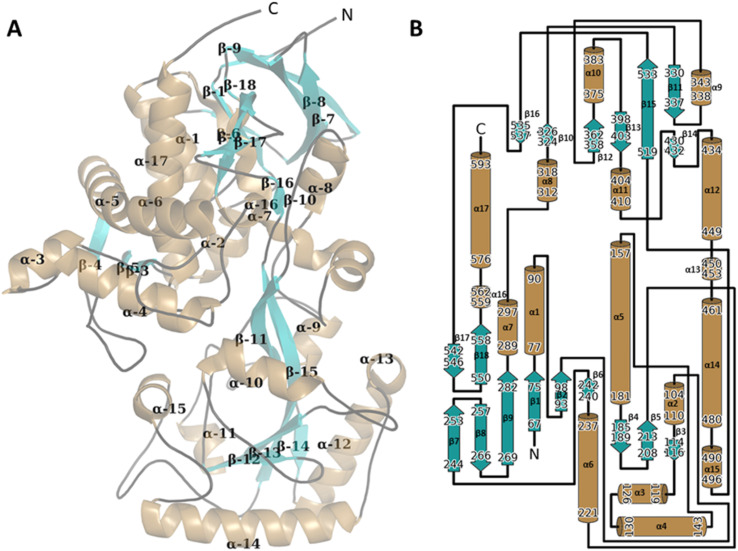
The 3D structure of the WT CDH monomer with the secondary structure manually annotated on to the structure (A). The structure was presented using PyMol (The PyMOL Molecular Graphics System, Version 1.2r3pre, Schrödinger, LLC). Alpha helices are mapped in sand, Beta sheets in teal and linker sequenced traced in black. The FAD ligand has been hidden for clarity. The secondary structure was assigned using PDB SUM http://www.ebi.ac.uk/thornton-srv/databases/pdbsum/ (B).

**Fig. 4:**
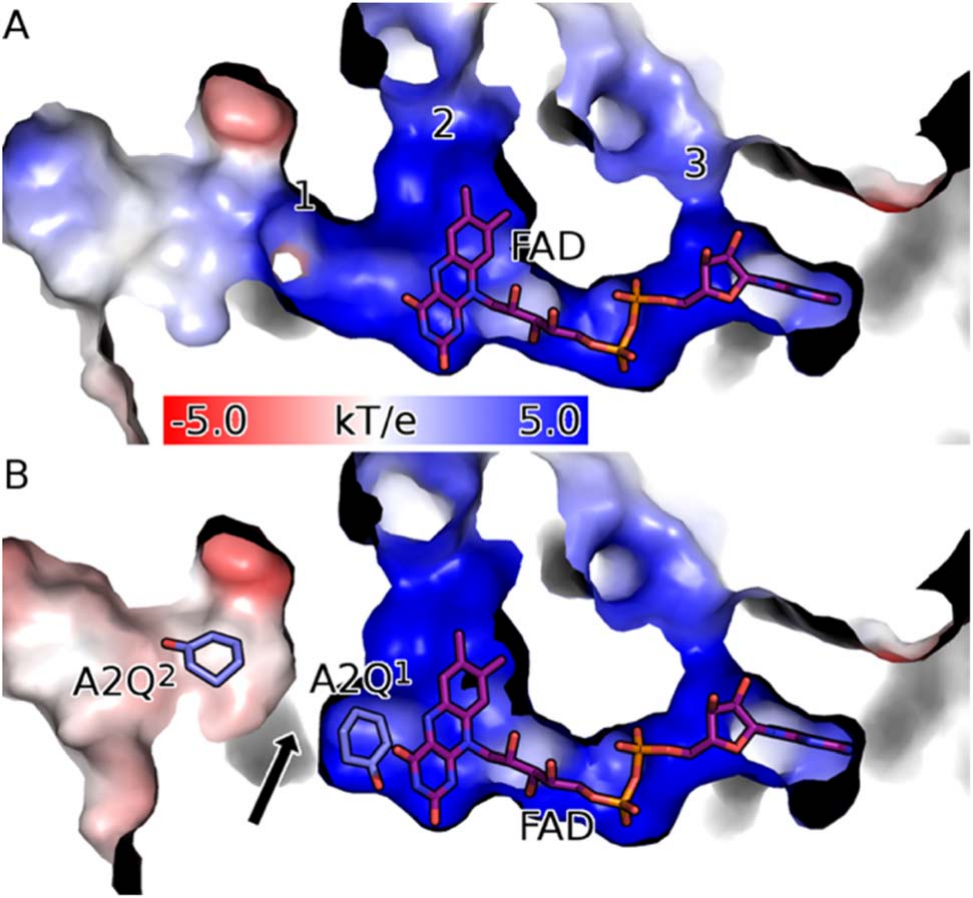
CDH binds FAD in a deep positively charged pocket within its FAD binding domain (F_d_). (A) The FAD (purple sticks) binding channel of the apo form of CDH (surface coloured according to electrostatic potential) has three solvent-accessible channels (labelled 1 to 3). Channel 1 is the route into the active site and is reached *via* a positively charged (blue surface) funnel. Channel 2 is filled with well-structured waters and is partially obscured by FAD, meaning it is too narrow to allow the passage of the substrate. Channel 3 exposes the ribose moiety of FAD to the solvent and is unlikely to be functional. (B) Upon binding the substrate in the active site (labelled A2Q^1^; mauve sticks) channel 1 of CDH Y195F closes (black arrow). Structures were drawn with PyMOL (DeLano Scientific https://www.pymol.org).

The SelenoMet WT CDH crystal structure was solved using single wavelength anomalous diffraction (SAD), using the selenium atom as the dispersion centre. The crystal structure of WT CDH is organised into two domains: a catalytic domain (C_d_) which is situated at the ‘top’ of the enzyme when viewed in the orientation shown in Fig. 3A and an FAD binding domain (F_d_) which is situated at the ‘bottom’. The C_d_ arises as an outcropping of the F_d_ α9–β11 loop and superficially resembles a Rossmann fold consisting of an α–β–α sandwich but consisting of just four β strands rather than the canonical six (Fig. 3A). The protein consisted of 4 sheets, 1 α–β–α unit, 6 β-hairpins, 8 β-bulges, 18 strands, 17 helices, 12 helix–helix interactions, 53 β-turns and 5 gamma turns (Fig. 3B). According to the PDBeFold structural database the closest structural homologue of C_d_ is the KSTD1 from *Rhodococcus erythropolis*, (4c3x – *Q* = 0.23; RMSD = 2.97), which itself is a member of the succinate dehydrogenase/fumarate reductase flavoprotein superfamily (Fig. S6†). The F_d_ domain is a classic example of a Rossmann fold. The FAD cofactor was found to occupy a deep positively charged channel through the interior of the F_d_ (Fig. 4) in agreement with other published structures.^30^ This pocket has three solvent-accessible channels: only one of these channels (labelled ‘1’ in Fig. 4A; this channel will subsequently be referred to as the entrance) is situated adjacent to the isoalloxazine moiety and is wide enough to allow passage of the substrate. This channel takes the form of a wide funnel formed between the F_d_ and catalytic C_d_ domains and opens into the active site (Fig. 4A). Channel 2 (subsequently referred to as the exit) is also adjacent to the isoalloxazine but partially occluded by it, meaning it is too narrow to allow passage of the substrate (Fig. 4A). It is however filled along its length with well-structured waters. The isoalloxazine is co-ordinated by the Sγ of Cys305 and the distance between Sγ of Cys305 and C8M of FAD is only 1.84 Å (Fig. S7†). There is density bridging this intermolecular gap, indicating a degree of shared electrons (Fig. S7†). The final channel, channel 3, is adjacent to the ribosyl moiety of the adenine nucleotide and is too narrow to allow even the passage of water (Fig. 4A). It can be seen with 2-cyclohexen-1-one in the active site, the entrance of the Y195F variant closes (Fig. 4B).

The asymmetric unit (ASU) for both the WT and the Y195F variant complexed enzymes contain two protein molecules bound in a head-to-head conformation (Fig. S8A and B†). The surface interface between the two molecules is ∼1970 (apo) and ∼1900 (holo) Å^2^. Analysis of these interfaces using PISA gives a solvation free energy gain (Δ^i^*G*) of −9.6 (apo) and −7.4 (holo) kcal mol^−1^, which suggests that these dimers are physiological and not crystallographic artefacts. The α10 loop (res 375–383) from each monomer is shared particularly intimately and forms extensive polar contacts with both the corresponding loop of the other monomer (Fig. S8C†). The orientation of the monomers relative to one another present access to the active sites on opposite sides of the dimer.

There is a high degree of similarity between all the protein molecules in the apo and holo ASUs of the Y195F variant (average pairwise Cα RMSD of 0.30 Å). The two apo monomers are highly similar, with a Cα RMSD of 0.07 Å. There is perhaps surprisingly less variation between the apo- and holo-enzymes than there is between the holo monomers (average Cα pairwise RMSD of 0.35 Å compared to 0.36 Å). Much of the variation between the apo- and holo-enzymes is accounted for by subtle interdomain shifts that occur during ligand binding (Fig. 4): the average pairwise Cα RMSDs between the apo and holo F_d_ and C_d_ domains is 0.24 Å and 0.28 Å, respectively.

#### Structure of the Y195F variant complex

**Fig. 5:**
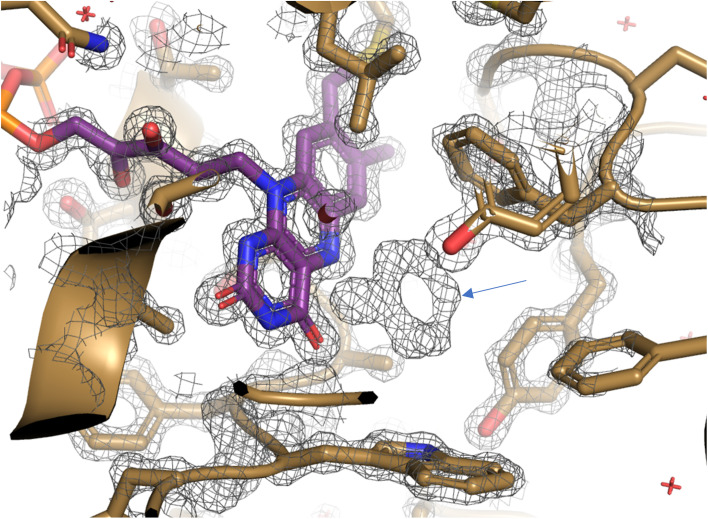
Unbiased map of cyclohexanone in complex with CDH·Y195A. Map (grey mesh) was refined in the absence of substrate and clearly shows unexplained density (blue arrow) in CDH active site (gold cartoon) adjacent to FAD (purple sticks). Map was refined using REFMAC5 and is shown at 2.5*σ* and visualised using PyMOL.

Initial refinement of the amino acid chains and FAD cofactors against the Y195F density map, revealed strong residual electron density in the putative active sites (Fig. 5). Upon evaluation this was found to be entirely consistent with the structure of 2-cyclohexen-1-one, the reaction product, and not the cyclohexanone substrate as expected. 2-cyclohexen-1-one was subsequently modelled to produce the Y195F complex structure. The occurrence of the product rather than substrate in the “inactive” mutant may be due to residual enzymatic desaturation and modification to phenol via tautomerisation. The Y195F complex was refined to the higher final resolution of 1.33 Å, compared to WT CDH (1.86 Å). An 11 amino acid stretch at the N terminus, that was disordered in the wild-type form, was found to be structured in the complex form. Significantly more structured waters were identified and refined in the complex form (1037 as opposed to 570). There is a funnel leading to the active site (Fig. S9A†) and three molecules of the product 2-cyclohexen-1-one (abbreviated using the PDB designation A2Q^1–3^), resulting from oxidation of the substrate cyclohexanone, were found in association with each protein molecule (Fig. S9B†). One of these (labelled A2Q^3^ in Fig. S9B†) was found bound in a shallow pocket on the surface of the protein distal from the active site and is unlikely to be of functional importance. A2Q^1^ is bound to the isoalloxazine moiety of FAD in a catalytically active conformation (Fig. S9B and C†).

#### Active site architecture

**Fig. 6:**
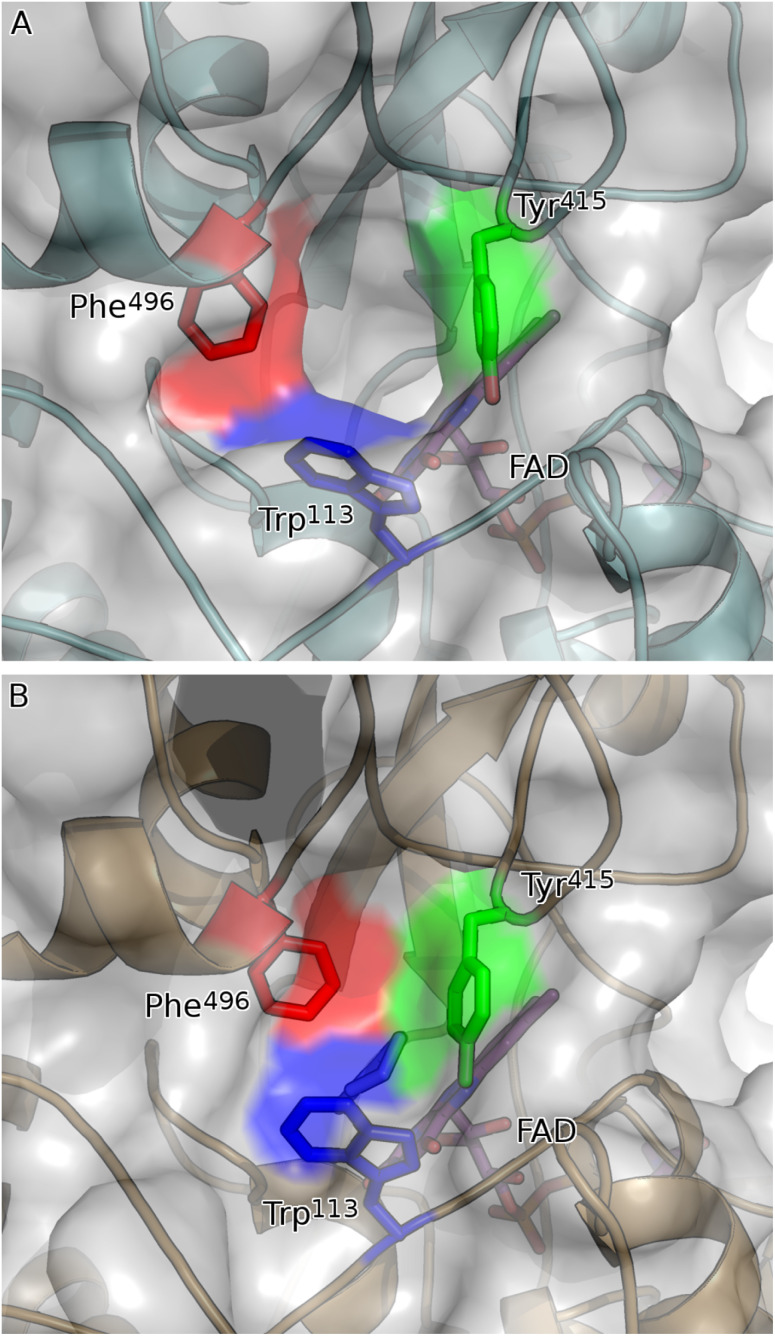
Network of aromatic residues co-ordinate to close active site upon binding of substrate. (A) Substrate binding pocket of apo form of CDH (teal cartoon) is ringed by three aromatic residues: Trp^113^ (blue sticks); Tyr^415^ (green sticks); and Phe^496^ (red sticks). These residues are in their open conformation, leaving a solvent-exposed channel into the active site of CDH. (B) Upon binding of 2-cyclohexen-1-one (mauve sticks), Trp^113^, Tyr^415^ and Phe^496^ close the active site of the holo form of CDH (gold cartoon) like the neck of a drawstring bag. Shown in (A) is chain A of the asymmetric unit: chain B has a bound molecule of PEG in the place of 2-cyclohexen-1-one and is intermediate between the open and closed forms (not shown).

The active site of the Y195F variant exists as a small chamber formed between the domains and contains four catalytic residues: Tyr^195^ (substituted by Phe in this study), Tyr^376^, Tyr^570^ and Gly^574^. The isoalloxazine moiety of FAD is positioned at the very base of the active site pocket, presenting its *re* face to the active site interior. Access to the active site is *via* a short positively charged passage (the entrance; Fig. 4A), which is itself accessed by a wide positively charged funnel. In addition to this channel, the active site is connected to the exterior *via* another narrower channel filled with well-structured waters (the exit; Fig. 4A). The orientation of the substrate is stabilised by hydrogen bond formation between the ketone group of the substrate and the amide group of Gly^574^ and the hydroxyl group of Tyr^570^. Upon substrate binding, three residues (Trp^113^, Tyr^415^ and Phe^496^), that form the mouth of the active site clamp shut, sealing the substrate in the active site (Fig. 6). This leaves the substrate packed into a pocket little bigger than itself and may help maintain the substrate in an optimal orientation for catalysis.

### Molecular dynamics simulations

**Scheme 2:**
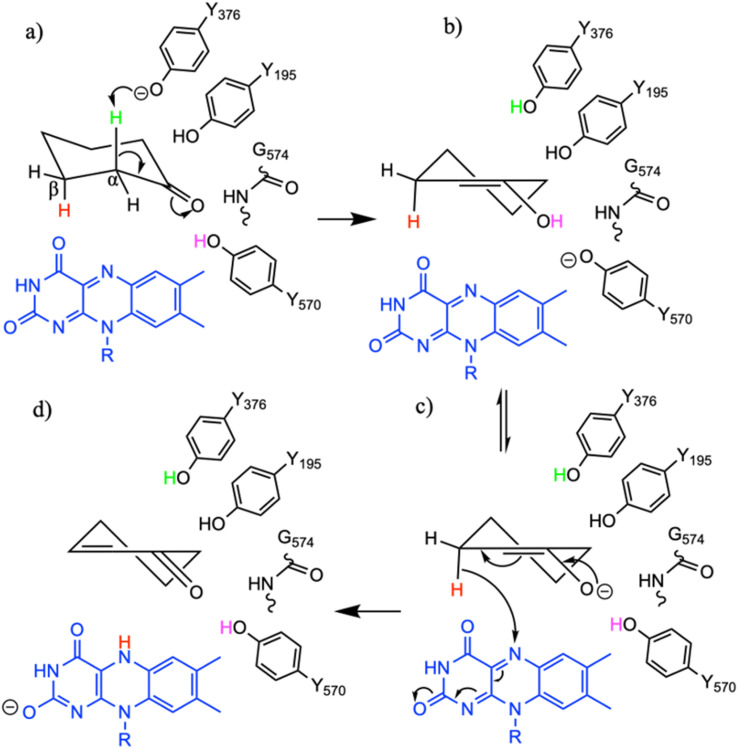
The proposed reaction mechanism of the oxidation of cyclohexanone by CDH enzymes. (a) Michaelis complex with cyclohexanone, (b) enol and enolate intermediate (c) formed after the axial proton abstraction from the alpha carbon atom of the cyclohexanone, (d) product cyclohexen-2-one formed after the hydride shift from the beta carbon of the enolate intermediate to the nitrogen atom of the FAD molecule.

To study the conformational dynamics of the WT CDH enzyme with its native substrate cyclohexanone, MD simulations were performed for 500 ns. Since the X-ray structure was the Y195F variant, the active site was reconstructed to Y195 in the model. MD simulations of the enzyme substrate complex (average RMSD value 1.2 Å, Fig. S10†) suggests that overall, there were no significant conformational changes in comparison to the X-ray structure. The cyclohexanone was stabilised very well in the active site of CDH and showed limited deviation from the X-ray structure, evidenced by a RMSD value of 0.2 Å (Fig. S11†). In the active site, cyclohexanone is in the hydrophobic pocked created by the W113, L530 and F352 residues. The cyclohexanone was also stabilised in the active site by π–π-interactions with the isoalloxazine of the FAD molecule. The carbonyl group of the cyclohexanone makes hydrogen bonds with the side chain of Y570 and the backbone of G574 (Fig. S9C and S11†). The Y376 or Y195 residues, which can potentially act as a catalytic base to initiate the dehydrogenation reaction, are in the vicinity of the axial proton (2.7 Å) located at the Cα position of the cyclohexanone (Fig. S9C and 7, see Fig. S12 for distances†). The orientation of cyclohexanone agrees with the X-ray structure of the Y195F variant and provides a productive model for catalysis (Fig. S9† and 7). The N5 atom of FAD, which can accept a hydride ion from the Cβ of cyclohexanone, is also at an acceptable distance of 2.8 Å of the Cβ of cyclohexanone (Fig. 6). Therefore, based on the productive Michaelis complex obtained from the Y196F variant X-ray structure and the MD simulations, we propose the cyclohexanone oxidation by CDH enzyme as shown Scheme 2. The Y376 anion can act as a catalytic base to initiate the axial proton abstraction of the Cα of cyclohexanone to form either enol or enolate intermediate (Scheme 2a). The negatively charged enolate intermediate in the active site of CDH is stabilised by the hydrogen bonds between the side chain of Y570 and the backbone of G574 residues (Scheme 2b and c). This is followed by the hydride shift from the Cβ of the enolate intermediate to the nitrogen atom of the FAD molecule to form the final product, cyclohexene-2-one (Scheme 2d). A similar reaction mechanism was reported in the recent computational and experimental study of the regioselective Δ^1^-dehydrogenation of the bulky 17-methyl-testosterone and dihydrotestosterone steroids by KSTD from *Rhodococcus erythropolis*.^6^ The reaction in the active site of KSTD is initiated by the proton abstraction from the steroid molecule by the tyrosyl anion followed by the hydride transfer to the FAD molecule.^5,6^

**Fig. 7:**
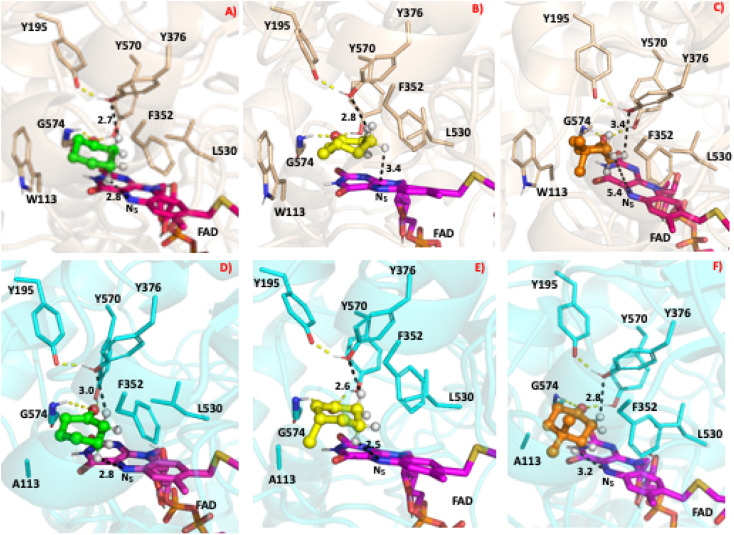
The most populated snapshot obtained using cluster analysis from the equilibrated MD trajectory of various ligands in complex with the WT-CDH and W113A mutant. The 3D structure of WT-CDH and W113A mutant were drawn in ribbon representation in wheat and cyan colours respectively. The cyclohexanone, 3-methylcyclohexanone and 4,4-dimethylcyclohexanone structures are shown in the CPK representation in green, yellow and orange colours respectively. Only the hydrogen atom on the alpha and beta position are shown for clarity purposes. (A) WT-CDH in complex with cyclohexanone, (B) WT-CDH in complex with 3-methylcyclohexanone, (C) WT-CDH in complex with 4,4-dimethylcyclohexanone, (D) W113A-mutant in complex with cyclohexanone, (E) W113A-mutant in complex with 3-methylcyclohexanone, (F) W113A-mutant in complex with 4,4-dimethylcyclohexanone. All the structure figures were drawn with PyMOL (DeLano Scientific https://www.pymol.org).

Experimental data suggest that 3-methylcyclohexanone and 4,4-dimethylcyclohexanone substrates also exhibits enzyme activity in CDH (Fig. S4†). Since there was no enzyme substrate complex available for these substrates, we docked these substrates into the active site of CDH. MD simulations were then performed to study their binding conformations in the active site of CDH. The 3-methylcyclohexanone and 4,4-dimethylcyclohexanone exhibit a similar conformation to cyclohexanone in the active site (Fig. 7). The axial proton of the Cα atom is 3.5 Å and 3.4 Å away from residue Y376, respectively. The axial hydride located on the and Cβ is on 2.7 Å and 5.4 Å away from the N5 atom of FAD, respectively. These distances indicate a productive model for catalysis. Overall, MD simulations of the WT CDH in complex with various ligands represent a productive structure to undergo dehydrogenation reaction (Scheme 2 and Fig. 7). The X-ray structure and the MD simulations presented us with the information needed to perform rational design of WT CDH. To make the CDH enzyme accept bulkier substrates, we envisioned the variants of the hydrophobic pocket which consists of residues W113, L530 and F352. To our delight, the W113A variant allowed the CDH enzyme to accept bulker substrates and improve the enzyme activity in comparison to WT CDH. To study how the W113A variant made the CDH enzyme more promiscuous, we performed MD simulations of the W113A variant in complex with native substrate cyclohexanone, 3-methylcyclohexanone and 4,4-dimethylcyclohexanone (Fig. 7). The MD simulations revealed that the W113A variant contains additional space in the active site to accommodate the methyl groups in the 3- and 4-substituted cyclohexanones and the fused aromatic ring of dihydrocoumarin.

## Conclusions

CDH offers a greener and more sustainable solution to the challenging α,β-desaturation of cyclic ketones. We have used both experimental and computational methods to elucidate the mode of action of CDH on various substituted cyclohexanones, lactones and lactams. The high-resolution *de novo* X-ray structure of the CDH in complex with cyclohexanone displayed the active site residues involved in substrate binding and catalysis. The in-depth study of WT-CDH using both biochemical assays and MD simulation allowed for the rational engineering of the CDH enzyme. The W113A variant showed overall improved activity and substrate scope. The experimental and computational data on CDH enzyme presented in the study provides a framework for rational protein engineering for efficient α,β-desaturation of cyclic ketones, lactones and lactams for the synthesis of important steroids and bioactive molecules.

## Data availability

Structural data is available in the pdb. Accession codes are given in Table 1. The secondary structure of the WT CDH monomer was assigned using PDB SUM (http://www.ebi.ac.uk/thornton-srv/databases/pdbsum/).

## Author contributions

Warispreet Singh - formal analysis, resources, visualization, writing – original draft, writing – review & editing. Nicola L. Brown - investigation. Hannah V. McCue – investigation. Sophie R. Marriott - investigation. Justin Perry - funding acquisition, writing – review & editing. Richard C. Wilson – investigation. Johan P. Turkenburg – investigation. Kshatresh D. Dubey – formal analysis, writing – review & editing. Stephen H. Prior – formal analysis, visualization, writing – original draft, writing – review & editing. Andrew J. Carnell - funding acquisition, resources, supervision, writing – review & editing. Edward J. Taylor – investigation, funding acquisition, resources, supervision, visualization, writing – original draft, writing – review & editing. Gary W. Black – conceptualization, funding acquisition, project administration, resources, supervision, writing – original draft, writing – review & editing.

## Conflicts of interest

There are no conflicts to declare.

## Supplementary Material

SC-015-D3SC04009G-s001

SC-015-D3SC04009G-s002

SC-015-D3SC04009G-s003

SC-015-D3SC04009G-s004

SC-015-D3SC04009G-s005

SC-015-D3SC04009G-s006

SC-015-D3SC04009G-s007

SC-015-D3SC04009G-s008

SC-015-D3SC04009G-s009

SC-015-D3SC04009G-s010

SC-015-D3SC04009G-s011

SC-015-D3SC04009G-s012

SC-015-D3SC04009G-s013
