# Supplementary material for: Rational design of a cyclohexanone dehydrogenase for enhanced α,β-desaturation and substrate specificity
Source: Chem Sci. 2024 Feb 21;15(13):4969–80. doi: 10.1039/d3sc04009g (PMC10966990; doi:10.1039/d3sc04009g)
Supplement: SC-015-D3SC04009G-s002 [file SC-015-D3SC04009G-s002.pdf]

Electronic Supplementary Material (ESI) for Chemical Science.
This journal is © The Royal Society of Chemistry 2024

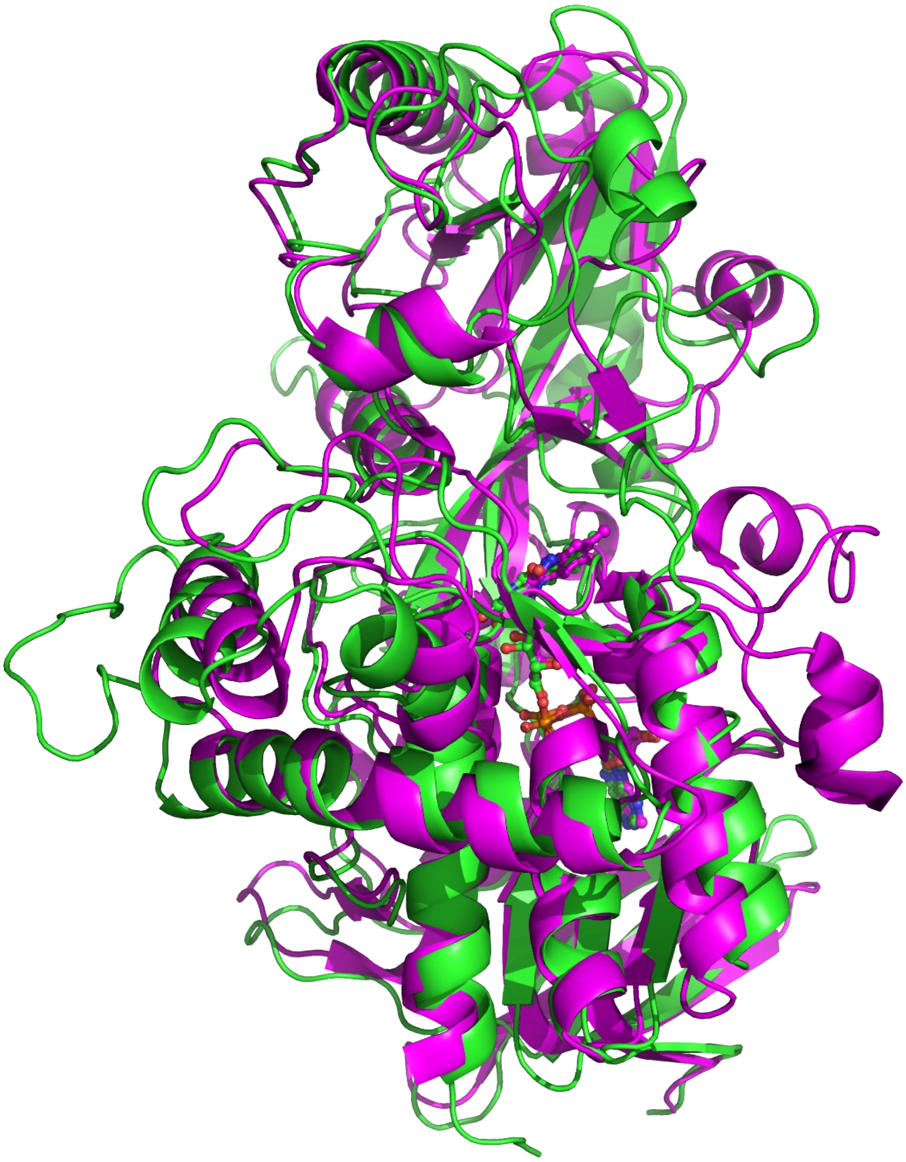

Figure S6. Superimposition of 3-Ketosteroid Δ1-Dehydrogenase from *Rhodococcus erythropolis* SQ1, PDB 4c3x(*1*) with wildtype CDH using COOT(*2*). rmsd achieved: 1.8823Å. The overlaid structures were drawn using PyMol.

1. A. Rohman, N. van Oosterwijk, A.-M. W. H. Thunnissen, B. W. Dijkstra, Crystal Structure and Site-directed Mutagenesis of 3-Ketosteroid Δ1-Dehydrogenase from Rhodococcus erythropolis SQ1 Explain Its Catalytic Mechanism*. *Journal of Biological Chemistry* **288**, 35559-35568 (2013).
2. P. Emsley, K. Cowtan, Coot: model-building tools for molecular graphics. *Acta Crystallogr D Biol Crystallogr* **60**, 2126-2132 (2004).
