# Supplementary material for: Rational design of a cyclohexanone dehydrogenase for enhanced α,β-desaturation and substrate specificity
Source: Chem Sci. 2024 Feb 21;15(13):4969–80. doi: 10.1039/d3sc04009g (PMC10966990; doi:10.1039/d3sc04009g)
Supplement: SC-015-D3SC04009G-s003 [file SC-015-D3SC04009G-s003.pdf]

Electronic Supplementary Material (ESI) for Chemical Science.
This journal is © The Royal Society of Chemistry 2024

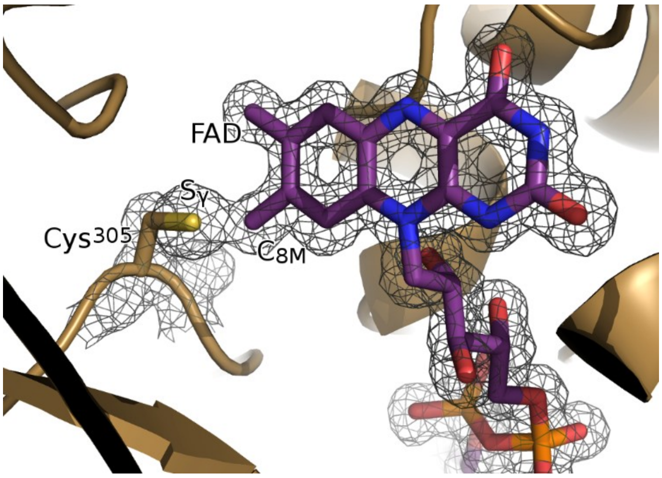

Figure S7. FAD (*purple* sticks) and CDH (*gold* cartoon) form an unusually short intermolecular contact. The S_γ atom of Cys^{305} (*gold sticks*) is situated only 1.84 Å from the C_{8M} atom of FAD. Electron density at 2.0 σ (*black mesh*) bridges this gap, indicating a degree of shared electrons. The complexed form is shown; the intermolecular distance was identical in the non-complexed form.
