# Supplementary material for: Rational design of a cyclohexanone dehydrogenase for enhanced α,β-desaturation and substrate specificity
Source: Chem Sci. 2024 Feb 21;15(13):4969–80. doi: 10.1039/d3sc04009g (PMC10966990; doi:10.1039/d3sc04009g)
Supplement: SC-015-D3SC04009G-s004 [file SC-015-D3SC04009G-s004.pdf]

Electronic Supplementary Material (ESI) for Chemical Science.
This journal is © The Royal Society of Chemistry 2024

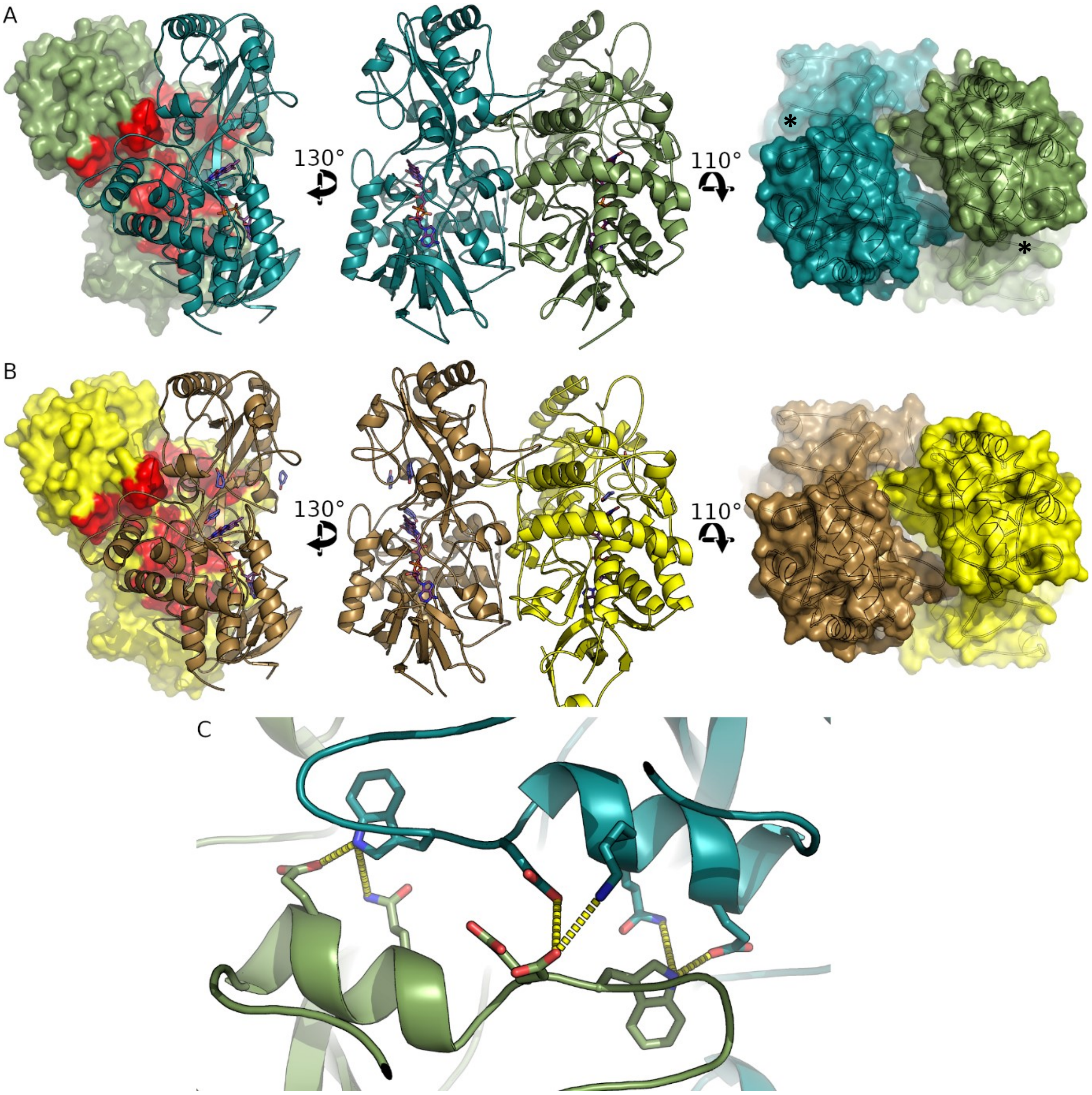

Figure S8. Dimer orientation in CDH. **Panel A.** Dimer orientation in WT apo enzyme. Chain A is shown as *teal cartoon* in standard orientation; chain B is shown as *green surface* with intermolecular interface shown in *red*. Monomers orient in head-to-head conformation. Approximate position of dimer active sites shown as asterisks. **Panel B**. CDH Y195F mutant in complex with cyclohexanone, surface representation as in A. Chain A shown in *gold*; chain B shown in *yellow* with interface shown in *red* **Panel C**. The α10 loop res (375-383) showing numerous intermolecular polar contacts. Residues forming hydrogen bonds shown as *sticks*; hydrogen bonds shown as *yellow dashed lines*.
