# Supplementary material for: Rational design of a cyclohexanone dehydrogenase for enhanced α,β-desaturation and substrate specificity
Source: Chem Sci. 2024 Feb 21;15(13):4969–80. doi: 10.1039/d3sc04009g (PMC10966990; doi:10.1039/d3sc04009g)
Supplement: SC-015-D3SC04009G-s005 [file SC-015-D3SC04009G-s005.pdf]

Electronic Supplementary Material (ESI) for Chemical Science.
This journal is © The Royal Society of Chemistry 2024

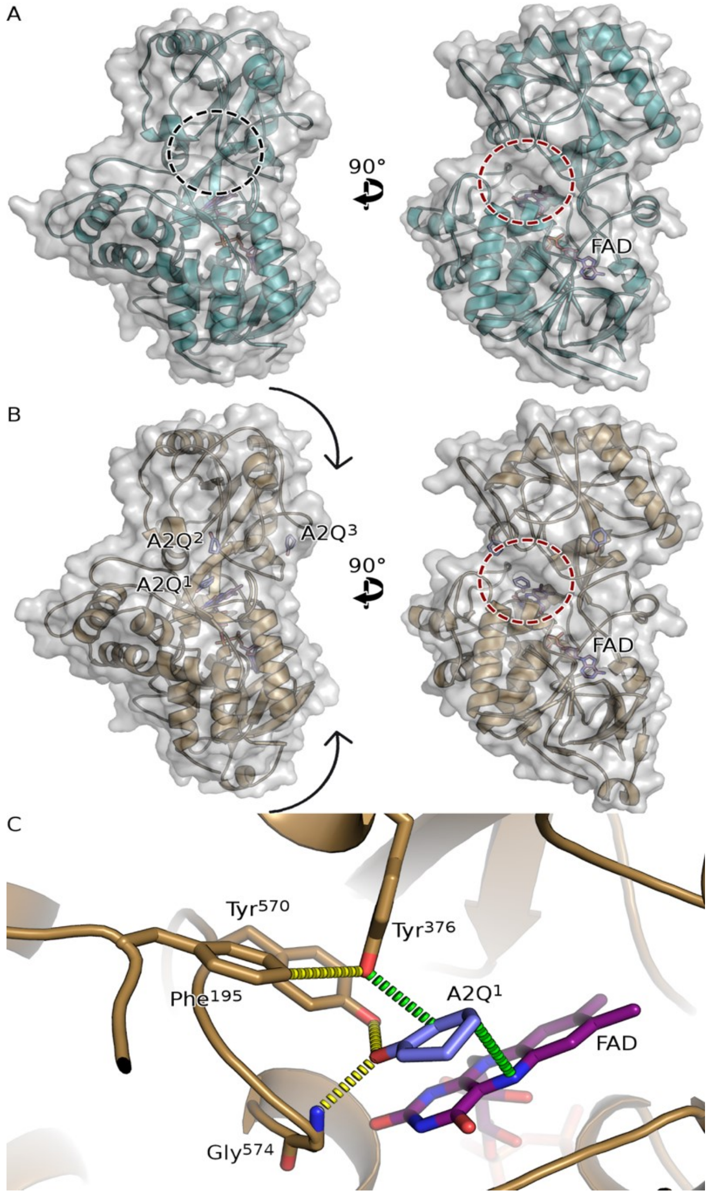

Figure S9. CDH and CDH-Y195F·A2Q have been solved to atomic resolution **A.** The apo form of CDH (*teal cartoon*) has a solvent-filled channel (the *entrance*) leading into the active site, which is formed as a cleft between the two domains. The inlet of the channel (*black dashed circle*) is wide enough to accommodate the passage of one molecule. The outlet of the channel (*red dashed circle*; the *exit*) is not wide enough to pass the substrate but is instead filled with well-structured waters, which possibly act as a reservoir of protons and electrons to reoxidise FADH (*purple sticks*) after catalysis. **B.** Upon binding three molecules (*mauve sticks*; labelled $A2Q^{1\text{-}3}$) the holo form of CDH Y195F (*gold cartoon*) undergoes subtle interdomain movement (indicated by *black arrows*). The binding of $A2Q^1$ into the active site triggers the closure of the inlet (Figure 2). $A2Q^2$ binds in the shallow pocket left after the closure of the inlet, but the occupancy is poor, and this binding event is unlikely to be functional. $A2Q^3$ binds in a shallow pocket distal from the active site, and is probably a crystallographic artefact. **C.** The active site of CDH Y195F with 2-cyclohexen-1-one bound. $A2Q^1$ stacks against the isoalloxazine ring of FAD, oriented in the correct position for dehydrogenation by the four catalytic residues (*gold sticks*) in concert with FAD. Hydrogen bonds are shown as *yellow dashed lines*; redox transfers are shown as *green dashed lines*. Structures were drawn with PyMOL (DeLano Scientific www.pymol.org).
