# Supplementary material for: Rational design of a cyclohexanone dehydrogenase for enhanced α,β-desaturation and substrate specificity
Source: Chem Sci. 2024 Feb 21;15(13):4969–80. doi: 10.1039/d3sc04009g (PMC10966990; doi:10.1039/d3sc04009g)
Supplement: SC-015-D3SC04009G-s006 [file SC-015-D3SC04009G-s006.pdf]

Electronic Supplementary Material (ESI) for Chemical Science.
This journal is © The Royal Society of Chemistry 2024

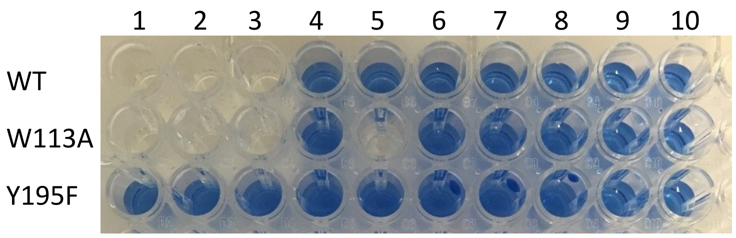

Figure S1A. DCPIP plate assay of CDH WT, W113A and Y195F enzyme variants with cyclohexanone (1), 3-methylcyclohexanone (2), 4,4-dimethylcyclohexanone (3), δ-valerolactone (4), dihydrocoumarin (5), 3,3,5-trimethylcyclohexanone (6), 1-methyl-2-piperidone (7), δ-valerolactam (8), 3,4-dihydro-2(1H)-quinolinone (9) and no substrate (10).

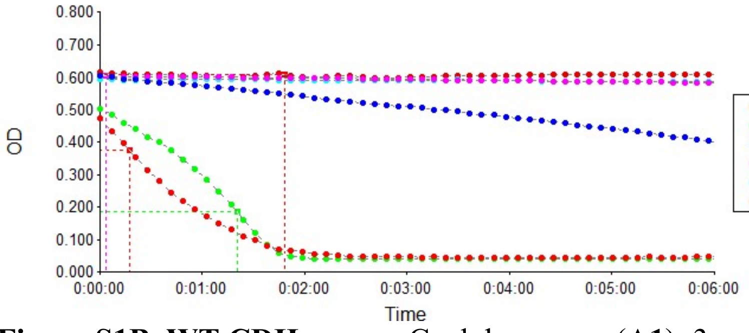

Figure S1B. WT CDH assays. Cyclohexanone (**A1**); 3-methylcyclohexanone (**A2**); 4,4-dimethylcyclohexanone (**A3**); δ-valerolactone (**A4**); dihydrocoumarin (**A5**); no substrate (**A10**). Time H:MM:SS.

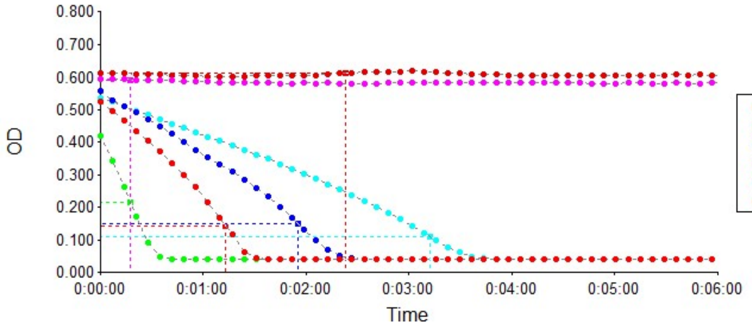

Figure S1C. W113A mutant assays. Cyclohexanone (**B1**); 3-methylcyclohexanone (**B2**); 4,4-dimethylcyclohexanone (**B3**); δ-valerolactone (**B4**); dihydrocoumarin (**B5**); no substrate (**B10**). Time H:MM:SS.

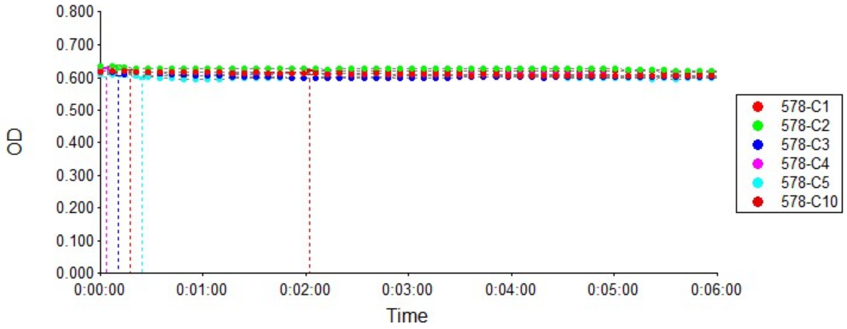

Figure S1D. Y195F mutant assays. Cyclohexanone (**C1**); 3-methylcyclohexanone (**C2**); 4,4-dimethylcyclohexanone (**C3**); δ-valerolactone (**C4**); dihydrocoumarin (**C5**); no substrate (**C10**). Time H:MM:SS.
