# Supplementary material for: Rational design of a cyclohexanone dehydrogenase for enhanced α,β-desaturation and substrate specificity
Source: Chem Sci. 2024 Feb 21;15(13):4969–80. doi: 10.1039/d3sc04009g (PMC10966990; doi:10.1039/d3sc04009g)
Supplement: SC-015-D3SC04009G-s007 [file SC-015-D3SC04009G-s007.pdf]

Electronic Supplementary Material (ESI) for Chemical Science.
This journal is © The Royal Society of Chemistry 2024

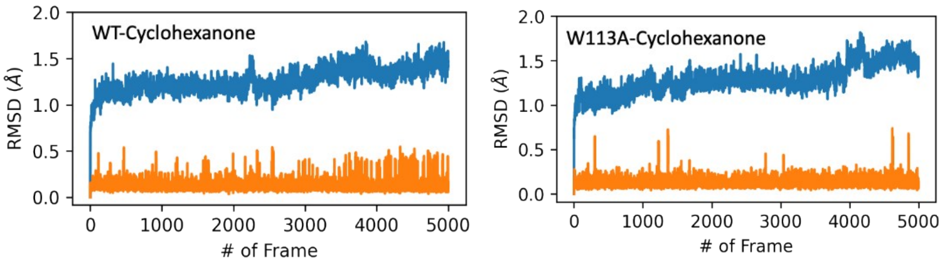

Figure S10. The RMSD obtained from wild type CDH and W113A in complex with cyclohexanone ligand for 500 ns trajectory. The RMSD values of Cα atoms of the protein and the heavy atoms of cyclohexanone ligand are shown in blue and orange colours respectively.
