# Supplementary material for: Rational design of a cyclohexanone dehydrogenase for enhanced α,β-desaturation and substrate specificity
Source: Chem Sci. 2024 Feb 21;15(13):4969–80. doi: 10.1039/d3sc04009g (PMC10966990; doi:10.1039/d3sc04009g)
Supplement: SC-015-D3SC04009G-s008 [file SC-015-D3SC04009G-s008.pdf]

Electronic Supplementary Material (ESI) for Chemical Science.
This journal is © The Royal Society of Chemistry 2024

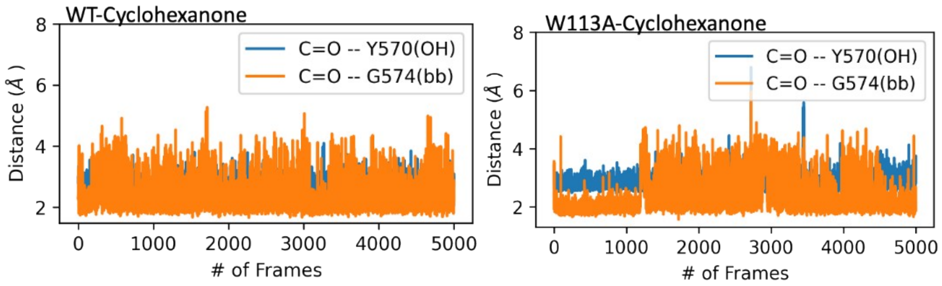

Figure S11. The hydrogen bonding in the wild type CDH and W113A in complex with cyclohexanone. The interatomic distance between the carbonyl oxygen of the cyclohexanone and the side chain of Y570 and the backbone of G574 residue for the 500 ns trajectory.
