# Supplementary material for: Rational design of a cyclohexanone dehydrogenase for enhanced α,β-desaturation and substrate specificity
Source: Chem Sci. 2024 Feb 21;15(13):4969–80. doi: 10.1039/d3sc04009g (PMC10966990; doi:10.1039/d3sc04009g)
Supplement: SC-015-D3SC04009G-s010 [file SC-015-D3SC04009G-s010.pdf]

This journal is © The Royal Society of Chemistry 2024

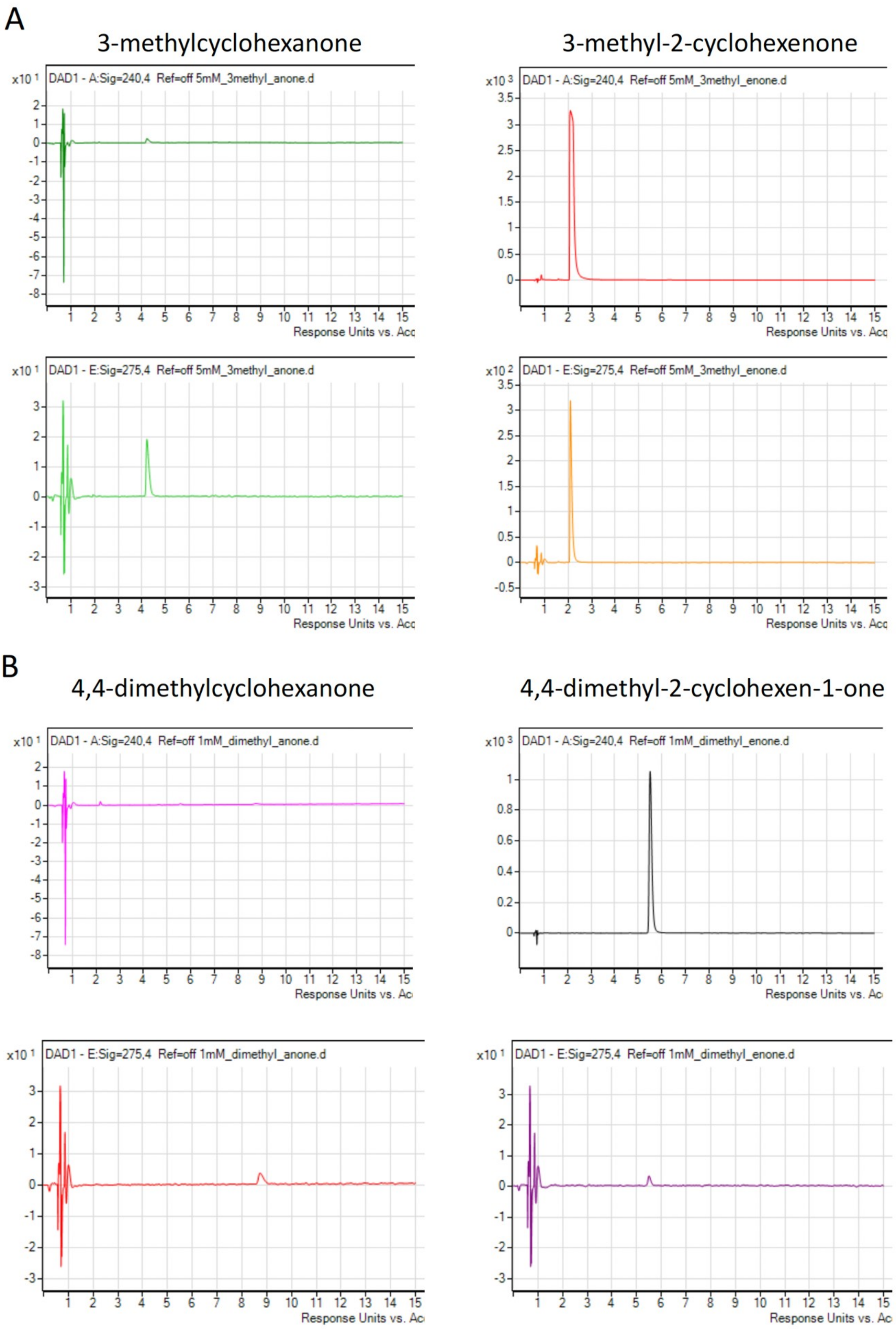

Figure S2. HPLC chromatograms from 5 mM standards of substrates (left) and corresponding products (right). Chromatograms were recorded at 240 nm (top) and 275 nm (bottom) for each standard. Chromatograms were acquired for 15-20 minutes. Substrates generally absorbed weakly at 275 nm and products absorbed much more strongly at 240 nm by virtue of their double bonds. 3-methylcyclohexanone had a retention time of ~4.2 min and 3-methyl-2-cyclohexenone had a retention time of ~2.0 min (**A**). 4,4-dimethylcyclohexanone had a retention time of ~8.8 min and 4,4-dimethyl-2-cyclohexen-1-one had a retention time of ~5.6 min (**B**).
