# Supplementary material for: Rational design of a cyclohexanone dehydrogenase for enhanced α,β-desaturation and substrate specificity
Source: Chem Sci. 2024 Feb 21;15(13):4969–80. doi: 10.1039/d3sc04009g (PMC10966990; doi:10.1039/d3sc04009g)
Supplement: SC-015-D3SC04009G-s011 [file SC-015-D3SC04009G-s011.pdf]

Electronic Supplementary Material (ESI) for Chemical Science.
This journal is © The Royal Society of Chemistry 2024

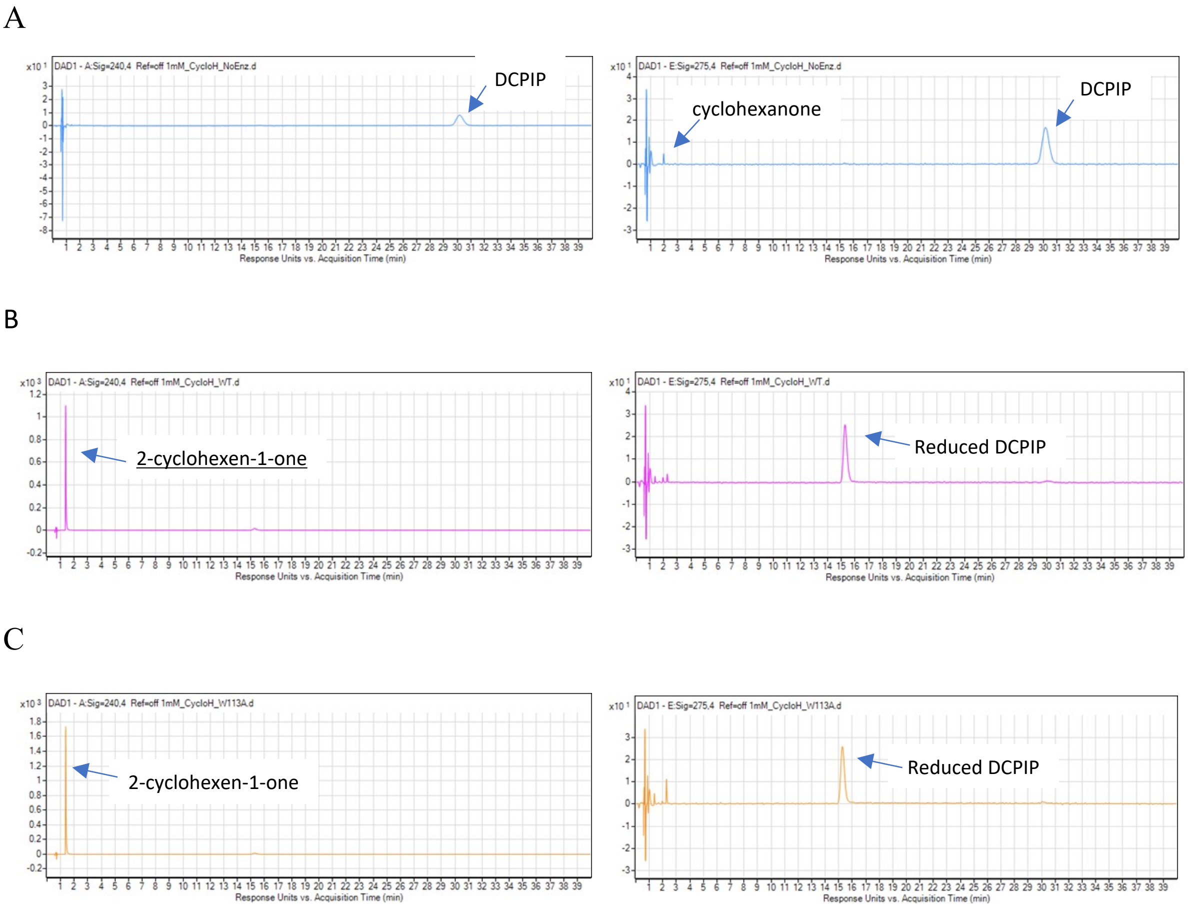

Figure S3. HPLC chromatograms showing incubation of cyclohexanone with DCPIP and no enzyme (**A**), WT CDH enzyme (**B**) and the W113A variant (**C**). Chromatograms were recorded at 240 nm and 275 nm. HPLC analysis of cyclohexanone incubated with DCPIP without enzyme revealed a peak for cyclohexanone at a retention time of 2 minutes and a peak for DCPIP at ~30.1 min. When cyclohexanone was incubated with CDH WT or the W113A variant, the DCPIP was reduced and therefore a change of retention time was observed from ~30.1 min to ~15.2 min and a peak for 2-cyclohexen-1-one was visible at ~1.4 min, confirming product formation. All retention times were compared with the retention times of the standards (Figure S2).
