# Supplementary material for: Rational design of a cyclohexanone dehydrogenase for enhanced α,β-desaturation and substrate specificity
Source: Chem Sci. 2024 Feb 21;15(13):4969–80. doi: 10.1039/d3sc04009g (PMC10966990; doi:10.1039/d3sc04009g)
Supplement: SC-015-D3SC04009G-s012 [file SC-015-D3SC04009G-s012.pdf]

Electronic Supplementary Material (ESI) for Chemical Science.
This journal is © The Royal Society of Chemistry 2024

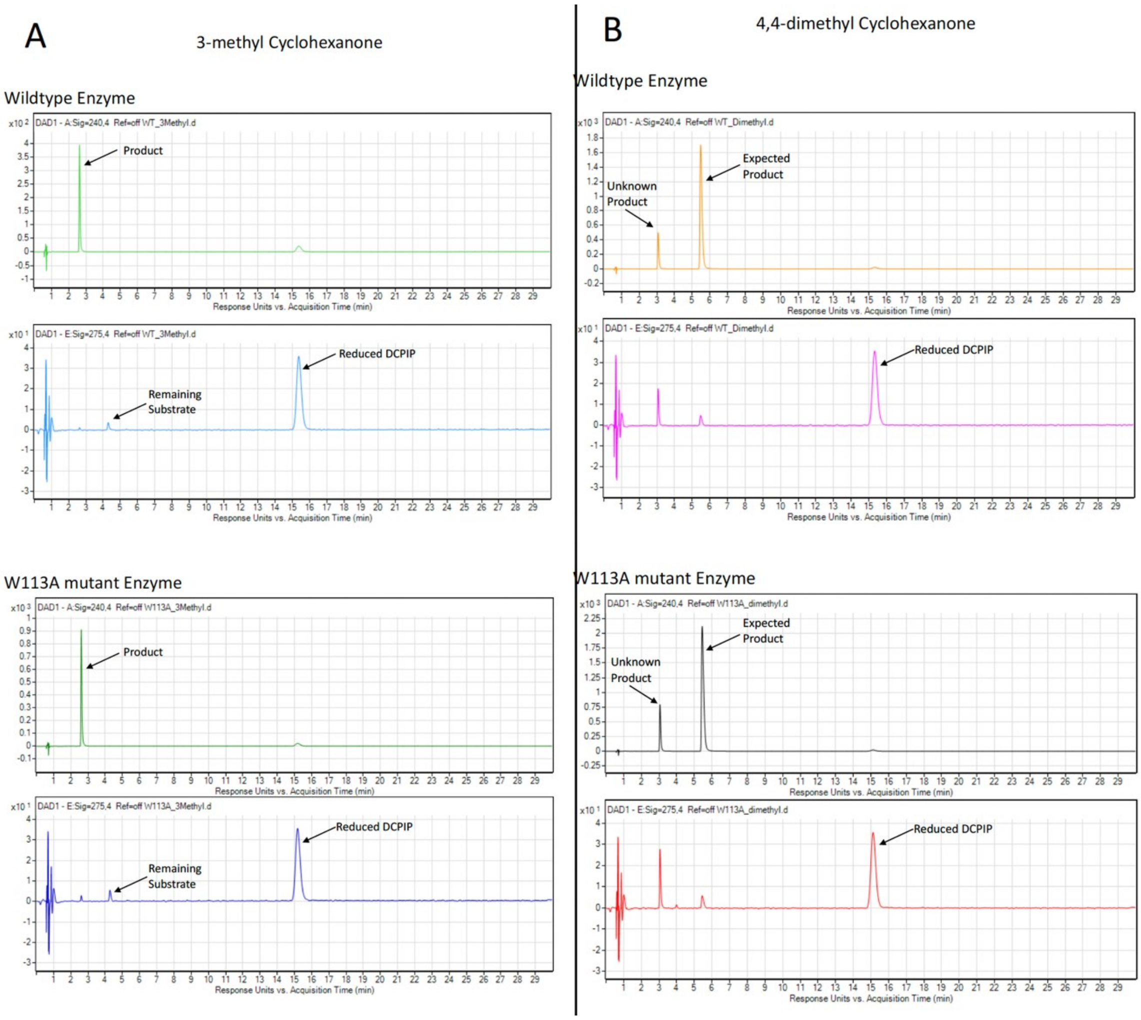

Figure S4. Example HPLC chromatograms showing products of 3-methylcyclohexanone (**A**), 4,4-Dimethylcyclohexanone (**B**) with either WT CDH or the W113A variant. Chromatograms were recorded at 240 nm and 275 nm. All retention times were compared with the retention times of the standards (Figure S2).
