# Supplementary material for: Rational design of a cyclohexanone dehydrogenase for enhanced α,β-desaturation and substrate specificity
Source: Chem Sci. 2024 Feb 21;15(13):4969–80. doi: 10.1039/d3sc04009g (PMC10966990; doi:10.1039/d3sc04009g)
Supplement: SC-015-D3SC04009G-s013 [file SC-015-D3SC04009G-s013.pdf]

Electronic Supplementary Material (ESI) for Chemical Science.
This journal is © The Royal Society of Chemistry 2024

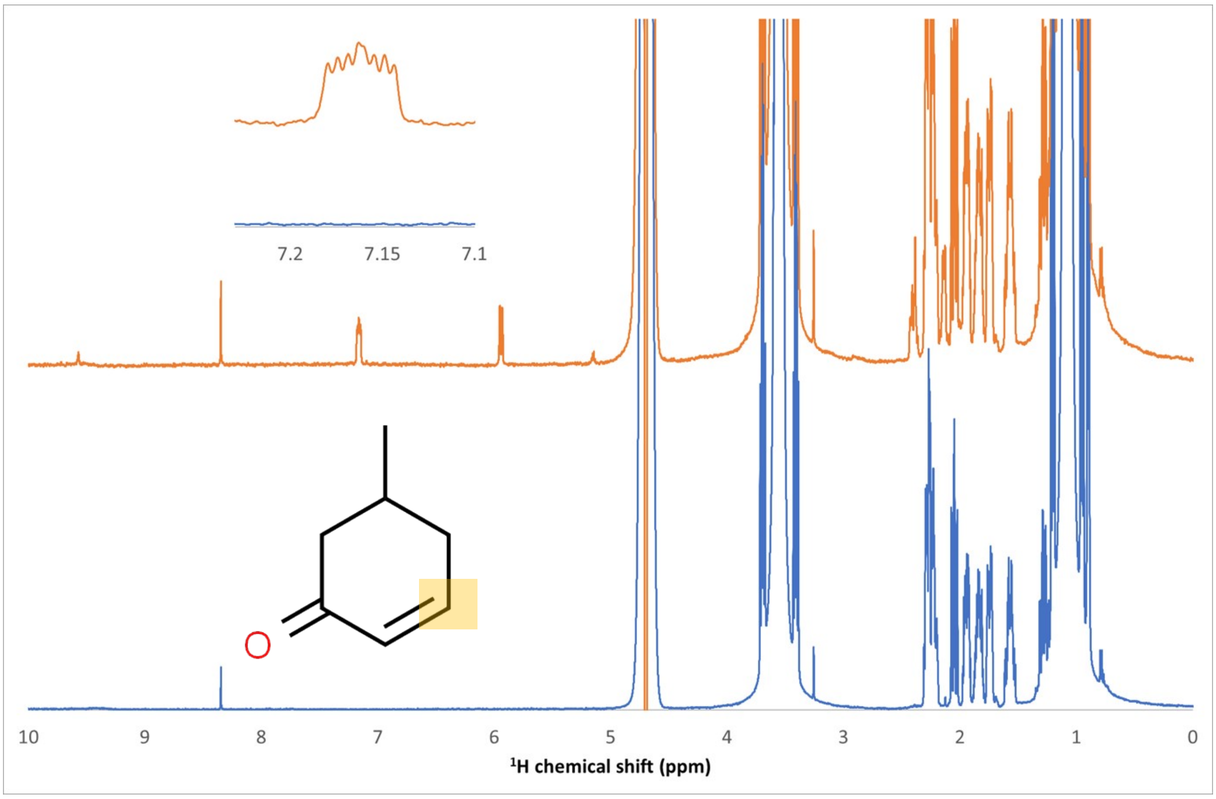

Figure S5. CDH catalyses the production of 5-methyl-2-cyclohexen-1-one. ¹H NMR spectra of 26 nM CDH (red; top trace) and 26 μM cold-denatured CDH (blue; bottom trace) incubated with 1 mM 3-methylcyclohexanone for 1 hour at 25°C. Inset shows detail of feature at c. 7.15 ppm, which corresponds to the proton highlighted in the structure of 5-methyl-2-cyclohexen-1-one. This feature is entirely absent in the cold-denatured spectrum. It has the characteristic ddd (*J* (Hz) = 9.93, 5.83, 2.96) shape expected for the 5-methyl isomer and would be absent in the case that CDH catalysed the production of the 3-methyl isomer.
